# The association of depression with child abuse among Indonesian adolescents

**DOI:** 10.1186/s12887-020-02218-2

**Published:** 2020-06-27

**Authors:** Meita Dhamayanti, Anindita Noviandhari, Nina Masdiani, Veranita Pandia, Nanan Sekarwana

**Affiliations:** 1 Department of Child Health, Faculty of Medicine, Hasan Sadikin Hospital, Universitas Padjadjaran, Jalan Pasteur No. 38, Pasteur, Sukajadi, Kota Bandung, Jawa Barat 40161 Indonesia; 2 Department of Psychiatry, Faculty of Medicine, Hasan Sadikin Hospital, Universitas Padjadjaran, Jalan Pasteur No. 38, Pasteur, Sukajadi, Kota Bandung, Jawa Barat 40161 Indonesia

**Keywords:** Adolescents, Child abuse, Depression, Indonesia

## Abstract

**Background:**

Depression is one of the most prevalent mental health problems among adolescents. Mental health problems might be the result of child abuse considering that their prevalences are increasing simultaneously in Indonesia. The aim of this study was to determine the association between depression and a history of abuse among adolescents.

**Methods:**

An analytic cross-sectional study was conducted on 786 junior high school students from Bandung City, West Java, Indonesia. Subjects were selected using two-stage cluster sampling. The Children’s Depression Inventory (CDI) and the ISPCAN Child Abuse Screening Tool (ICAST) questionnaires were applied to assess depression and a history of abuse, respectively. Depression was diagnosed by a psychiatrist after a positive score on the CDI. The data were analysed using chi-square tests and multiple regression.

**Results:**

A history of child abuse was associated with depression in adolescents. All dimensions of child abuse had a significant association with depression. Psychological violence had the highest risk factor for the occurrence of depression (PR = 6.51), followed by exposure to violence and physical violence. Sexual violence was not a common dimension of child abuse among students. Psychological violence had the strongest association with depression, and victims were three times more likely to develop depression (POR = 3.302, *p* = 0.004).

**Conclusion:**

Psychological violence was proven to be a strong risk factor for developing depression symptoms among adolescent students. While each victimization domain remained a significant predictor of depression, the experience of multiple domains during a child’s life-course may predict mental health risk. Early detection and interventions to prevent abuse and its consequences are critical.

## Background

Child abuse includes all forms of physical and mental child abuse, sexual abuse, neglect or negligent treatment, and commercial or other exploitation that has a high likelihood of resulting in actual or potential harm to the child’s health, survival, development, dignity, responsibility, beliefs and/or rights [1–3]. The prevalence of child abuse is increasing [4]. Meta-analyses have provided a series of overall estimations of 17.7, 26.7, 11.8 and 16.3% for physical abuse, psychological abuse, sexual abuse, and neglect, respectively [5–7]. The Indonesian Commission of Child Protection [8] reported an increase in violence from 2.178 cases in 2011 to 6.006 in 2015. In terms of the setting of violence, 91% occurred at home, 87.6% at school, and 17.9% in the community [8].

A history of violence against children might lead to mental health disorders, such as depression, psychosis, anxiety and post-traumatic disorder [9, 10]. Depression is one of the mental health problems that commonly occur in adolescents. Currently, approximately 2 to 3% of children and 8% of adolescents have experienced depression. The lifetime prevalence of depressive disorders in adolescents is estimated to be 17% [11].

Adolescents with depression may become a burden on their families and themselves. The impact of depression is not only detrimental to the child and family but also a national burden [12]. Adolescents with depression may have a higher risk of decline in academic performance, interpersonal relations, and suicide [13]. A history of child abuse at the age of 10 to 17 years is the strongest predictor of depression, adolescents who experience violence in school and at home have the highest risk of depressive disorders in society [14, 15]. In most cases, child abuse by a caregiver or parent at home is the form of victimization that has the strongest independent association with depression [14].

West Java is one of thirty-four provinces in Indonesia. It has a 9.3% prevalence of mental emotional problems in adolescents above 15 years of age while Indonesia’s national rate is at 6% [15]. Accordingly, this research aimed to analyse the relationship between a history of child abuse and depression in adolescents in West Java, Indonesia.

## Methods

### Study design

A cross-sectional study was conducted on junior high school students. A two-stage cluster sampling was performed to determine the school and the subjects. First, several schools were selected randomly, and then the adequate number of students was determined by a simple random sampling method. A minimum sample size of 770 students was needed in this study (99% power and 95% significance interval). The study was conducted from May to December 2016.

A letter of approval from the Provincial Directorate of National Education of the city where the research took place was obtained prior to the study. Ethical approval was issued by the Health Research Ethics Committee Faculty of Medicine, Universitas Padjadjaran 29/UN6.C1.3.2/KEPK/PN/2016.

### Tools

Depression was assessed using the Children’s Depression Inventory (CDI) [16, 17] as well as interviews for the application of a diagnosis based on the criteria of the DSM V diagnostic criteria. Depression criteria were determined by the answers to the questionnaire. Later, with the determination of a CDI score ≥ 19, an interview was carried out by a psychiatrist based on the DSM V diagnostic criteria. The CDI instrument itself has been validated in an Indonesian version [18].

To assess any history of child abuse, we used the ICAST-C questionnaire [19, 20],which has been validated in an Indonesian version [21]. The ICAST-C consists of 55 questions covering 5 dimensions of abuse: violence (9 questions), physical (19 questions), psychological (17 questions), neglect (6 questions), and sexual (4 questions). Scores for each question were interpreted as follows: 1 = if there is a history of violence and 0 = if there is no history of violence. A cut-off point was selected using the mean value of the history of child abuse data. Subjects with a total score of child abuse dimensions below the cut-off point were not categorized as experiencing child abuse.

For the correlation validity test, we compared the items correlation value with the reference value by taking 5% of the number of respondents (45) as the ɑ value. The result was 0.294. The results showed that all items in the ICAST-C had adequate validity. Based on the Kuder-Richardson reliability test method, the ICAST-C instrument showed strong reliability (KR20 = 0.92 and KR21 = 0.87).

### Data analysis

Descriptive tests with numeric and percentage value presentation were used to analyse the results of the CDI and I-CAST. Analytic Chi-square tests were used to analyse the differences of socio-demographic characteristics of students among subjects with depression and associations between the scores of both instruments. Bivariate analysis between the histories of child abuse with depression was tested with the Prevalence Ratio (PR). Multivariate tests were applied to determine which type of abuse was the most correlated with depression. The results were considered significant if the *p* value was < 0.05. Data management and analysis were conducted using SPSS (Statistical Package for Social Science) 15.0.

## Results

**Fig. 1:**
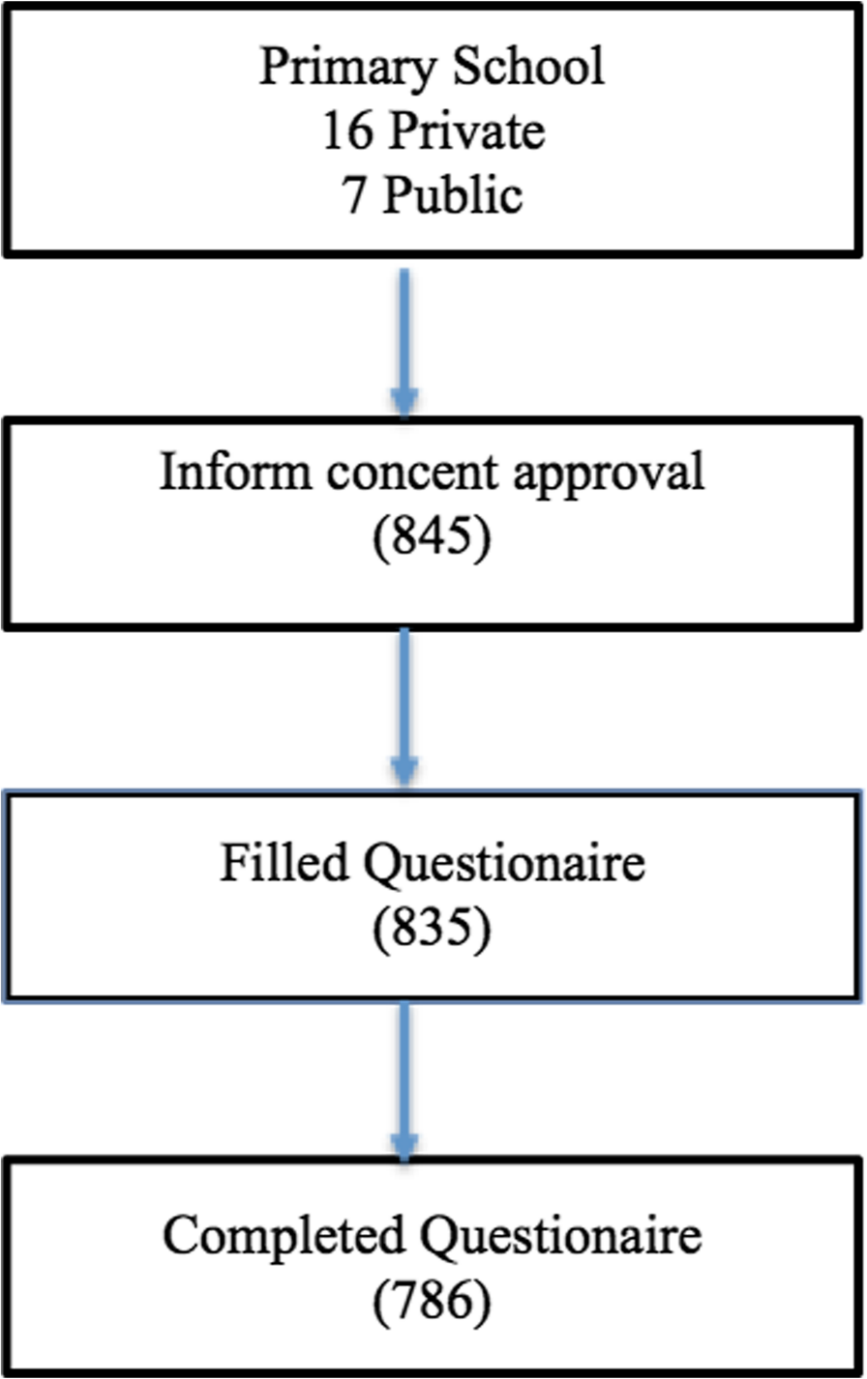
Sample Selection

A total of 845 students from 23 junior high schools gave their consent; however, only 835 filled out a questionnaire. After examining the questionnaire, 786 students participated in this study (Fig. 1). Students were from the 7th grade (34.86%), 8th grade (36.51%), and 9th grade (28.63%) of junior high school. There was an almost equal proportion of male and female students, at 441(56.11%) and 345 (43.89%), respectively. The age range was 12–16 years old, with a mean age of 13 years old. A total of 43 (5.47%) subjects with a CDI score ≥ 19 were subsequently interviewed by a psychiatrist and met the DSM-V criteria of depression. There were no significant differences of students’ socio-demographic characteristics among depressed and non-depressed subjects (Table 1).

**Table 1:** The socio-demographic comparison among depressed and non-depressed subjects

Characteristics	Depression		p
	No (***n*** = 743)	Yes (***n*** = 43)	
**Age**			0.168**
Mean	13.36	13.56	
SD	0.926	0.881	
Median	13.0	13.0	
Range	12–16	12–15	
	**n(%)**	**n(%)**	
**Sex**			
Male (*N* = 441)	420 (95.2)	21 (4.8)	0.323*
Female (*N* = 345)	323 (93.6)	22 (6.4)	
**Grade**			
7th (*N* = 274)	263 (96.0)	11 (4.0)	
8th (*N* = 287)	270 (94.1)	17 (5.9)	0.395*
9th (*N* = 225)	210((3.3)	15 (6.7)	
**Father’s Education**			
Primary School (N = 74)	70 (94.6)	4 (5.4)	
Junior High School N = (82)	79 (96.3)	3 (3.7)	
Senior High School (*N* = 300)	285 (95.0)	15 (5.0)	0.802*
College and higher (+ 324)	303 (93.5)	21 (6.5)	
Illiterate (*N* = 6)	6 (100.0)	0	
**Mother’s Education**			
Primary School (*N* = 75)	73 (97.3)	2 (2.7)	
Junior High School(*N* = 102)	100 (98.0)	2 (2.0)	
Senior High School (*N* = 329)	310 (94.2)	19 (5.8)	0.202*
College and higher (*N* = 272)	252 (92.6)	20 (7.4)	
Illiterate (*N* = 8)	8 (100.0)	0	
**Father’s Occupation**			
Public Servant (*N* = 295)	279 (94.6)	16 (5.4)	
Entrepreneur (*N* = 337)	321 (95.3)	16 (4.7)	0.556*
Labourers/Farmers/Others (*N* = 154)	143 (92.9)	11 (7.1)	
**Mother’s Occupation**			
Public Servant (*N* = 116)	107 (92.2)	9 (7.8)	
Entrepreneur (*N* = 145)	137 (91.9)	12 (8.1)	0.181*
Labourers/Farmers/Others (*N* = 54)	51 (94.4)	3 (5.6)	
Housewife (*N* = 467)	448 (95.9)	19 (4.1)	

*p* value using *Chi-square test and ** T test

A history of child abuse was determined by the statistical mean rate of the child abuse score. A subject with scores above the mean value was categorized as having a history of child abuse. There were 367 subjects (46.7%) with a history of child abuse, as shown in Table 2.

**Table 2:** Association between depression and child abuse history

Child abuse	Depression		p
	No (*n* = 743)	Yes (*n* = 43)	
**No** (*n* = 419)	403 (51.27%)	16 (2.04%)	0.030*
**Yes** (*n* = 367)	340 (43.26%)	27 (3.43%)	

*Chi-square *p* < 0.05

A total of 27 (3.43%) subjects with depression had an experience of child abuse. A significant association was found between depression and a child abuse history (*p* = 0.03) (see Table 2).

There were subjects with a history of psychological victimization (474, 60.3%), exposure to violence (354, 45%), physical victimization (321, 40.8%), neglect (313, 39.8%) and sexual victimization (172, 21.9) (Table 3). Based on bivariate analysis, all 5 dimensions of child abuse history were associated with depression. Subjects with a psychological victimization history were 6.51 times more likely to develop depression, while those with neglect were three times more likely to develop depression (Table 3).

**Table 3:** Association between depression and dimensions of child abuse

	Depression		*p*-value	PR	95% CI
Child abuse dimension	No n (%)	Yes n (%)			
Psychological victimization	438 (55.73)	36 (4.58)	0.00**	6.51	2.85–14.81
Violence exposure	322 (40.9)	32 (4.07)	0.00**	3.80	1.88–7.66
Physical victimization	291 (37.02)	30 (3.82)	0.00**	3.58	1.84–6.98
Neglect	285 (36.26)	28 (3.56)	0.00**	3.00	1.57–5.71
Sexual victimization	154 (19.59)	18 (2.29)	0.00**	2.75	1.46–5.18

Note: * Chi-square test; *PR* Prevalence risk

Since bivariate analysis had shown an association of all dimensions with depression, multivariate analysis was conducted to find the dimension with the strongest association with depression using logistic regression tests. The results shown in Table 4 indicate that psychological violence has the strongest association. Adolescents with a history of psychological violence were three times more likely to develop depression (POR = 3.302, *p* = 0.004).

**Table 4:** Multivariate regression between depression and child abuse dimensions

Variable	coeff B	SE (B)	p value	POR_**adj**_ (95% CI)
**First model:**				
Psychological	0.854	0.453	0.060	2.348 (0.966–5.708)
Violence exposure	0.902	0.427	0.035	2.464 (1.068–5.684)
Physical	0.463	0.391	0.237	1.589 (0.738–3.421)
Neglect	0.242	0.337	0.472	1.274 (0.658–2.466)
Sexual	0.437	0.342	0.202	1.548 (0.792–3.027)
**Last model:**				
Psychological	1.195	0.414	0.004	3.302 (1.466–7.438)
Violence exposure	1.096	0.415	0.008	2.993 (1.328–6.747)

## Discussion

This study revealed that child abuse was quite common among junior high school students in Bandung, West Java, Indonesia. Approximately 46.69% of our subjects had a history of child abuse. Indonesia itself has no definite data about child abuse so far. However, this current study showed a history of victimization in psychological dimensions was the most widely experienced by the students (60.3%), followed by exposure to violence (45%), physical victimization (40.8%) and neglect (39.8%). On the other hand, sexual victimization was relatively uncommon (21.88%).

Epidemiological data from a study in India showed that the highest prevalence of violence experienced was psychological (61.9%), physical (21.43%) and sexual (16.67%) [22]. Meanwhile, data from the United States for 686,000 cases of children violence showed that neglect was the most common (78.6%), followed by physical abuse (18.3%) and sexual violence (9.3%) [22]. However, several studies showed the cumulative prevalence based on a survey of communities were approximately 15–30% for girls and 5–15% for boys for sexual violence, 5–35% for physical violence, 4–9% for psychological violence and 6–12% for neglect [22].

Most of the subjects were seventh grade students who were in the early adolescent phase (ages 11 to 14) and were typically very egocentric with poor self-regulation [23]. These young adolescents were mostly emotionally victimized at school. School violence played an important role in developing depression [12, 24]. Meanwhile, conduct disorder has its highest prevalence in adolescents, namely, 7% in adolescents aged 12 to 16 years old [25].

This study showed all child abuse dimensions had significant associations with depression. This is similar to prior studies that asserted the existence of a correlation between histories of violence against children with depression [26, 27]. Therefore, while different forms of victimization frequently co-occur, they each make unique additional contributions towards an increased risk for mental health problems [14].

Subjects with a history of psychological child abuse had a 6.51 times higher risk of depression. This finding is similar to a meta-analysis that stated abuse and neglect were strongly associated with depressive disorder in adolescence [24]. Moreover, Pirdehghan’s study in Iran showed a correlation between mental disorders and violence (Spearman rho: 0.2; *p*-value < 0.001) as well [28].

We found that depressive symptoms on the CDI were strongly associated with all dimensions of child abuse, particularly psychological violence and neglect. In one review of 124 studies, psychological violence increased the risk of depression by an odds ratio of 3.06, whereas physical abuse increased the risk of depression by only half that amount. Furthermore, psychological violence abuse was more closely related to depression severity than sexual or physical abuse [10].

Emotional abuse and neglect may alter the development of reward and oxytocin systems in childrens’ brains, leading to impaired parental care giving in the subsequent generation [29].

These findings were confirmed in a recent systematic review and meta-analysis, although most of the data came from retrospective cross-sectional studies or longitudinal designs that relied on self-reported abuse [30]. Sexual violence showed a far weaker association. There are several possible explanations, such as that it may be underreported because of stigma [31].

Practitioners should be aware that violence during childhood might result in negative consequences in adolescents. Therefore, a good understanding is required to prevent violent acts against children that might allow effective interventions into violence issues in adolescents [32, 33].

## Conclusions

A history of child abuse has a correlation with depression in adolescents. Psychological child abuse had the highest risk for the onset of depression compared to other violent dimensions. However each victimization domain remained a significant predictor of depression, the experience of multiple domains during a child’s life-course may predict mental health risk. Early detection and interventions to prevent abuse and its consequences are critical.

## Limitations

We recognized that a diagnosis of depression might be over-represented by the adolescent students fulfilling the questionnaires. To better reflect the accuracy of this diagnosis, psychiatrists were used to determine those that met the DSM-V criteria for depression.

## Data Availability

All data and materials of this study are available at the Faculty of Medicine, Universitas Padjadjaran, Bandung, Indonesia, by contacting the corresponding author Meita Dhamayanti, email meita.dhamayanti@unpad.ac.id

## Abbreviations

CDI: Children’s Depression Inventory
ISPCAN: International Society for Prevention of Child Abuse and Neglect
ICAST: ISPCAN Child Abuse Screening Tool
DSM-V: Diagnostic and Statistical Manual of Mental Disorders V
ICAST-C: ISPCAN Child Abuse Screening Tool Children’s Version
KR: Kuder-Richardson
SPSS: Statistical Package for the Social Sciences
PR: Prevalence Ratio
POR: Prevalence Odds Ratio
